# Recombinant Human Soluble Thrombomodulin Suppresses Monocyte Adhesion by Reducing Lipopolysaccharide-Induced Endothelial Cellular Stiffening

**DOI:** 10.3390/cells9081811

**Published:** 2020-07-30

**Authors:** Takayuki Okamoto, Eiji Kawamoto, Haruki Usuda, Tetsuya Tanaka, Tetsuro Nikai, Kunihiro Asanuma, Koji Suzuki, Motomu Shimaoka, Koichiro Wada

**Affiliations:** 1 Department of Pharmacology, Faculty of Medicine, Shimane University, 89-1 Enya-cho, Izumo-city, Shimane 693-8501, Japan; h-usuda@med.shimane-u.ac.jp (H.U.); tetsu@med.shimane-u.ac.jp (T.T.); koiwada@med.shimane-u.ac.jp (K.W.); 2 Department of Molecular Pathobiology and Cell Adhesion Biology, Mie University Graduate School of Medicine, 2-174 Edobashi, Tsu-city, Mie 514-8507, Japan; a-2kawamoto@clin.medic.mie-u.ac.jp (E.K.); shimaoka@doc.medic.mie-u.ac.jp (M.S.); 3 Department of Emergency and Disaster Medicine, Mie University Graduate School of Medicine, 2-174 Edobashi, Tsu-city, Mie 514-8507, Japan; 4 Department of Human Nutrition, Faculty of Contemporary Life Science, Chugoku Gakuen University, 83 Niwase, Kita-ku, Okayama-city, Okayama 701-0197, Japan; 5 Department of Anesthesiology, Faculty of Medicine, Shimane University, 89-1 Enya-cho, Izumo-city, Shimane 693-8501, Japan; t.nikai@med.shimane-u.ac.jp; 6 Department of Orthopaedic Surgery, Mie University Graduate School of Medicine, 2-174 Edobashi, Tsu-city, Mie 514-8507, Japan; kasanum@gmail.com; 7 Faculty of Pharmaceutical Sciences, Suzuka University of Medical Science, 3500-3, Minamitamagaki-cho, Suzuka-city, Mie 513-8679, Japan; suzukiko@suzuka-u.ac.jp

**Keywords:** endothelial cells, cellular stiffness, thrombomodulin, cell adhesion, gap junctions

## Abstract

Endothelial cellular stiffening has been observed not only in inflamed cultured endothelial cells but also in the endothelium of atherosclerotic regions, which is an underlying cause of monocyte adhesion and accumulation. Although recombinant soluble thrombomodulin (rsTM) has been reported to suppress the inflammatory response of endothelial cells, its role in regulating endothelial cellular stiffness remains unclear. The purpose of this study was to investigate the impact of anticoagulant rsTM on lipopolysaccharide (LPS)-induced endothelial cellular stiffening. We show that LPS increases endothelial cellular stiffness by using atomic force microscopy and that rsTM reduces LPS-induced cellular stiffening not only through the attenuation of actin fiber and focal adhesion formation but also via the improvement of gap junction functionality. Moreover, post-administration of rsTM, after LPS stimulation, attenuated LPS-induced cellular stiffening. We also found that endothelial cells regulate leukocyte adhesion in a substrate- and cellular stiffness-dependent manner. Our result show that LPS-induced cellular stiffening enhances monocytic THP-1 cell line adhesion, whereas rsTM suppresses THP-1 cell adhesion to inflamed endothelial cells by reducing cellular stiffness. Endothelial cells increase cellular stiffness in reaction to inflammation, thereby promoting monocyte adhesion. Treatment of rsTM reduced LPS-induced cellular stiffening and suppressed monocyte adhesion in a cellular stiffness-dependent manner.

## 1. Introduction

Endothelial cells are a crucial player in the regulation of inflammation, blood coagulation, leukocyte adhesion, and vascular remodeling in inflamed vessels. The current consensus on vascular inflammatory diseases holds that endothelial cellular dysregulation occurs simultaneously in both inflammatory and coagulant systems. Thus, endothelial cell dysfunction is a cause of the vascular lesions associated with functional and structural abnormalities in vascular vessels. Thrombomodulin (TM) is an anticoagulant protein that is predominantly expressed on the surface of endothelial cells [1,2,3]. TM directly binds to thrombin and inhibits thrombin procoagulant activity [1,2]. The thrombin-TM complex alters thrombin substrate specificity and accelerates anticoagulant activated protein C generation [1,2]. Recent preclinical studies have documented the favorable effects of recombinant soluble TM (rsTM) in experimental sepsis, including vascular inflammation and blood coagulation state [4,5,6,7]. In addition to its anticoagulant properties, it is thought that rsTM has anti-inflammatory effects. In fact, the C-type lectin-like domain of TM inhibits an alamin high-mobility group box 1 (HMGB1) protein [8] and lipopolysaccharide (LPS) [9], and suppresses neutrophil adhesion to endothelial cells [10]. Subsequently, rsTM promotes the suppression of mitogen-activated protein kinase pathways and nuclear factor-κB activation [10]. However, the anti-inflammatory mechanism of rsTM remains only partially understood.

Recent studies have reported that the arterial stiffening observed during atherosclerosis is a cholesterol-independent risk factor for cardiovascular events [11]. Vascular stiffness has been primarily determined by the reconstitution of extracellular matrix components, fibrosis, and calcification in vascular vessels [12]. In addition, the increased stiffness of vascular smooth muscle cells and endothelial cells has similarly been observed in aging-related arterial stiffening and atherosclerotic plaques, respectively [13,14]. Our recent studies have also shown that endothelial cells increase their stiffness in response to proinflammatory stimuli [15]. Interestingly, leukocytes and platelets are more likely to adhere to stiff vascular vessels and substrates [16,17], suggesting that endothelial cellular stiffening may promote the formation of atherosclerotic plaques and thrombus via the enhancement of leukocyte and platelet adhesion to inflamed vascular endothelium.

Gap junctions are intercellular junctional channels composed of connexin (Cx) protein subunits that allow direct cell–cell communication by transfer ions, amino acids, small metabolites, and secondary messengers to adjacent cells [18]. In addition to intercellular communication through this channel function, the intracellular domain of Cx proteins promotes cellular signaling via interactions with intracellular phosphatases and protein kinases, catenins, structural proteins, and microtubules [19]. Thus, gap junction-mediated endothelial cellular communication is involved in the regulation of endothelial cell functions. It has been known that gap junction-mediated endothelial cellular communication is attenuated by proinflammatory and procoagulant stimuli [20]. Abnormal expression of endothelial Cxs and loss of gap junction function have been associated with the onset of cardiovascular diseases [21,22,23]. Of note, the enhancement of leukocyte adhesion and transmigration during atherogenesis has been observed in endothelial Cx-deficient mice [23,24]. Our previous studies demonstrated that the inhibition of gap junction functionality promotes endothelial cellular stiffening, suggesting the possibility that the proinflammatory stimulation-induced cellular stiffening that occurs by reducing gap junction functionality in endothelial cells contributes to the promotion of leukocyte adhesion to inflamed endothelial cells [15].

In this study, we found that LPS-induced endothelial cellular stiffening facilitates monocyte adhesion. We determined that rsTM improves LPS-induced cellular stiffening through the suppression of actin fiber formation and the enhancement of gap junction function. In addition, we demonstrated that rsTM suppresses monocyte adhesion to endothelial cells in a cellular stiffness-dependent manner. Our results show that rsTM improves LPS-induced cellular stiffening and reduces gap junction functionality, leading to the suppression of monocyte adhesion to inflamed endothelial cells.

## 2. Materials and Methods

### 2.1. Cell Culture

Primary human umbilical vein endothelial cells (HUVECs) and culture media (EGM-2 BulletKit) were purchased from Lonza Japan (Tokyo, Japan). HUVECs were cultured in collagen-coated tissue-culture dishes (Corning, Corning, NY, USA) in an atmosphere containing 95% air and 5% CO_2_. HUVECs from different donors were used in each experiment. All experiments were performed with cultured endothelial cells during passages 3–5. An rsTM protein was provided by Asahi Kasei Pharma Corporation (Tokyo, Japan). To examine the effect of LPS from *Escherichia coli* on endothelial cellular stiffening, HUVECs were grown to confluency and stimulated with 1 μg/mL of LPS. The stiffness measurements of HUVECs were performed at two time points: 4 and 24 h after the addition of LPS (Figure S1A). In order to investigate the effect of rsTM on LPS-induced endothelial stiffening and rsTM dose dependency, HUVECs were treated with 1 μg/mL of LPS and TM at the indicated concentration for 4 h (Figure S1B,C). To test the effect of post-administration of rsTM, we stimulated HUVECs with LPS for 1 h, and then treated them with 10 μg/mL of rsTM for 3 h (Figure S1D).

### 2.2. Determination of Cellular Stiffness

**Figure 1:**
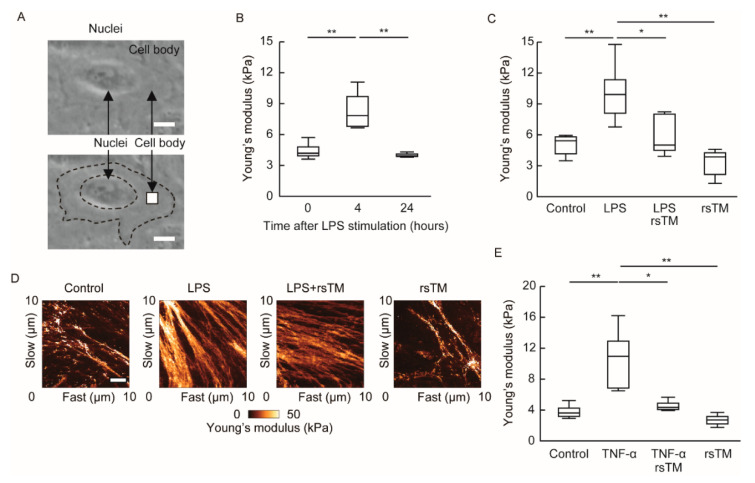
Measurements of cultured endothelial cellular stiffness after LPS stimulation and/or rsTM administration. (**A**) The scanning field indicated by the grey square (10 μm × 10 μm) is shown for a single cell. One area in the cell was measured and visualized. Scar bar showing 16 μm. (**B**) The stiffness of HUVECs after LPS stimulation. The Young’s modulus (kPa) of HUVECs at 0, 4, and 24 h after LPS stimulation are shown. Box plots range from the 25th to 75th percentiles, and the line inside the box represents the median (*n* = 4 cells at 0 h, *n* = 5 cells at 4 h, and *n* = 3 cells at 24 h). *p* values were determined by two-way ANOVA with Tukey’s test, ** *p* < 0.01. The experiments were repeated three independent times with similar results. (**C**) The stiffness of HUVECs stimulated with LPS and/or rsTM for 4 h was determined (*n* = 5 cells, each group). *p* values were determined by two-way ANOVA with Tukey’s test, * *p* < 0.05; ** *p* < 0.01. The experiments were repeated three independent times with similar results. (**D**) Stiffness images of HUVECs were obtained by AFM. Recorded Young’s moduli data at a resolution of 128 × 128 pixels in a scanning field of a cell were reconstructed as a stiffness image. The stiffer area in cells is showing as brighter. Representative images from five samples in Figure 1C are shown. Scar bar showing 2 μm. (**E**) HUVECs were stimulated by TNF-α (10 units/mL) with or without rsTM (10 μg/mL) for 4 h. After stimulation, the stiffness of HUVECs was determined by AFM. Box plots of the Young’s modulus of HUVECs (*n* = 4 cells per group) are shown. TNF-α stimulation increased cellular stiffness, whereas rsTM treatment significantly suppressed TNF-α-induced cellular stiffening. *p* values were determined using a two-way ANOVA with Tukey’s test, * *p* < 0.05; ** *p* < 0.01. The experiments were repeated three independent times with similar results.

Cellular stiffness, defined as the resistance from the deformation of the cell against applied force, was measured using the NanoWizard 3 AFM (atomic force microscopy) system (JPK Instruments AG, Berlin, Germany) as reported previously [15,25]. The basic principle of this method is to indent a cell with a cantilever and measure the force curves from the bending of the cantilever that occurred depending on the physical property of the cell. The Young’s modulus, which is a unit of cellular stiffness, is evaluated by fitting the curves of the measured force by AFM using the Hertz contact model. Briefly, cells were randomly selected from live HUVEC monolayers. Young’s moduli of cells were measured using an AFM with a cantilever and a tetrahedral-type probe (Olympus, Tokyo, Japan) at the indicated time points for a given condition. All force curves and scanning field images (10 μm × 10 μm) were recorded at a resolution of 128 × 128 pixels in quantitative imaging (QI) mode at 37 °C. The scanning field (10 μm × 10 μm) was captured in a single cell on the cell body, excluding the nucleus and cellular edges (Figure 1A). In order to determine the stiffness of a group, 3 to 6 different cells were measured. The data were processed by curve fitting with the Hertz contact model using JPK data processing software and shown as the stiffness image. The geometric mean of the Young’s modulus was calculated from the acquired Young’s modulus of the scanning field. After calculation, recording data at a resolution of 128 × 128 pixels in a cell was reconstructed as a stiffness image.

### 2.3. Fluorescent Imaging of F-Actin and Vinculin in HUVECs

To assess actin fiber and focal adhesion formation, actin and vinculin were visualized. Briefly, after stimulation with LPS and/or rsTM, HUVECs were fixed with 4% paraformaldehyde for 30 min, and then permeabilized with 0.05% tween20 in phosphate-buffered saline (PBS) for 30 min. After permeabilization, actins were visualized using rhodamine-phalloidin (Thermo Fisher Scientific, Waltham, MA, USA). Vinculin were stained with anti-human vinculin monoclonal antibody (Sigma, St. Louis, MO, USA) and Alexa488-conjugated anti-mouse IgG antibody (Thermo Fisher Scientific). Nuclei were stained using 4′,6-diamidino-2-phenylindole (DAPI) (Dojindo, Kumamoto, Japan). HUVECs were observed using Olympus widefield fluorescence microscopy. Images without irregular cells, noise, and/or debris were taken. The fluorescence intensity of phalloidin-stained images and the number of vinculin spots in a cell were analyzed by using ImageJ 1.53a software (US National Institutes of Health, Bethesda, MD).

### 2.4. Dye Transfer Assay

Gap junction functionality was assessed by a modified preloaded dye-transfer technique as follows [26]. Acceptor HUVECs were cultured in 24-well cell-culture plates to confluency. Donor HUVECs labeled with calcein-AM (Dojindo) and 4′,6-diamidino-2-phenylindole (DAPI) were then co-cultured for 2 h with the acceptor HUVECs. After 2 h, the plates were rinsed with PBS three times to remove floating donor HUVECs and background fluorescence. Migration of the dye through the gap junctions from dye-loaded donor HUVECs to neighboring acceptor HUVECs, nuclei of donor HUVECs stained by DAPI, and brightness image were all observed and counted using a fluorescence microscope. The field containing 10 to 20 donor cells was captured and analyzed.

### 2.5. THP-1 Cell Adhesion to Substrates

Intercellular adhesion molecule-1 (ICAM-1)-Fc-coated hydrogel and CellTiter-Glo (Promega, Madison, WI) were used to assess stiffness-dependent THP-1 cell adhesion. Briefly, Easy Coat gels (1, 4, 12, 25, or 50 kPa) in 24-well plates (Softwell; Matrigen, Brea, CA, USA) were coated with recombinant ICAM-1-Fc (5 μg/mL) or recombinant Fc (5 μg/mL) for 1 h and then blocked with bovine serum albumin (BSA) at 37 degrees for 2 h. Cells were diluted to a concentration of 5.0 × 10^5^ cells per mL in growth media and 500 μL of cell suspension were added to the gel in a 24-well plate. After 2 h of incubation, to remove non-adhesive cells, gels were twice washed with PBS. Cells bound to the coated gel were quantified by CellTiter-Glo according to the manufacturer’s instructions.

### 2.6. THP-1 Cell Adhesion to HUVECs

To prepare soft or stiff endothelial cells, HUVECs were treated with 10 μM of blebbistatin (Merck Millipore, Darmstadt, Germany) or 1 μM of carbenoxolone (Sigma). They were then stimulated by tumor necrosis factor-α (TNF-α) (10 units/mL, FUJIFILM Wako, Osaka, Japan) for 4 h. THP-1 cells were stained with calcein-AM and co-cultured with HUVECs for 1 h. After incubation, to remove non-adhesive cells, HUVECs were washed twice with PBS. THP-1 cells bound to HUVECs were counted using an Olympus IX71 fluorescence microscope.

To assess the effect of rsTM-mediated cellular softening on monocyte adhesion, HUVECs were treated with rsTM (0, 1, 3, 10 μg/mL) in the presence or absence of LPS (1 μg/mL) for 4 h. HUVECs were washed twice with PBS and then cocultured with HUVECs for 1 h. After incubation, to remove non-adhesive cells, HUVECs were washed twice with PBS. THP-1 cells bound to HUVECs were observed under an Olympus IX71 fluorescence microscope. Quantification of the THP-1 cells that had adhered to HUVECs was performed using ImageJ 1.53a software.

### 2.7. Statistical Analyses

Statistical analysis between data pairs was carried out using a Mann–Whitney U test. Differences between the groups were analyzed by using two-way analysis of variance (ANOVA) followed by Tukey’s test. Differences from negative controls were determined by using one-way ANOVA followed by Dunnett’s test. All of the statistical tests were performed using R software. Statistical significance was set to a *p* value less than 0.05. The specific and appropriate statistical tests performed are indicated in the figure legends.

## 3. Results

### 3.1. RsTM Reduces LPS-Induced Endothelial Cellular Stiffening

Our previous results demonstrated that proinflammatory TNF-α or thrombin stimulation transiently induces an increase in endothelial cellular stiffness after 4 h [15]. In addition to these proinflammatory stimuli, we examined whether LPS-mediated sterile inflammation induces endothelial cellular stiffening. To this end, we stimulated confluent HUVECs with LPS and then measured cellular stiffness at part (10 μm × 10 μm) of a cell body by using AFM after 4 and 24 h of stimulation (Figure 1A and Figure S1A). We randomly picked up the indicated number of cells and compared the cellular stiffness after LPS stimulation. Endothelial cells increased their stiffness after 4 h of proinflammatory LPS stimulation and returned to baseline levels at 24 h (Figure 1B). This alteration in cellular stiffness was similar to that observed in phenotypes after TNF-α or thrombin stimulation [15].

Although the anti-inflammatory and cytoprotective effects deployed by rsTM to counter inflamed endothelial cells have been known [3,5,6], the impact of rsTM on endothelial cellular stiffening under inflammatory conditions remains elusive. Therefore, we investigated whether rsTM suppresses endothelial cellular stiffening during LPS-mediated sterile inflammation. We simultaneously treated HUVECs with LPS and rsTM, and measured cell stiffness after 4 h (Figure S1B). In contrast to LPS stimulation, co-treatment of rsTM significantly suppressed LPS-induced endothelial cellular stiffening (Figure 1C). We confirmed that rsTM suppressed TNF-α-induced endothelial cellular stiffening (Figure 1E). These results indicated that rsTM attenuated endothelial cellular stiffness in response to inflammatory stimuli and not by blocking the interaction of LPS-Toll-like receptor 4. Figure 1D shows a stiffness image of the scanning area (10 μm × 10 μm) in a cell. The stiffer area, indicated as brighter, increased after LPS stimulation. Stiffness images showed the formation of rigid fiber-like regions in the LPS-treated group and reduced them in the LPS- and rsTM-treated group (Figure 1D). These results suggest that rsTM may reduce cellular stiffness as a consequence of attenuation of the actin structure.

**Figure 2:**
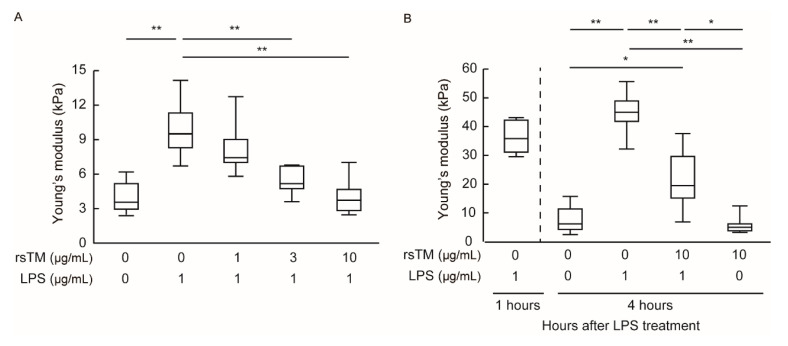
RsTM suppressed LPS-induced endothelial cellular stiffening. (**A**) The stiffness of LPS-stimulated HUVECs treated with rsTM at 0, 1, 3, 10 μg/mL for 4 h was measured. Young’s modulus was determined from the force curve (*n* = 5 cells, each group). *p* values were determined with a Dunnett’s test, ** *p* < 0.01 vs. group with 1 μg/mL of LPS and 0 μg/mL of rsTM. The experiments were repeated three independent times with similar results. (**B**) The stiffness of LPS-stimulated HUVECs after post-administration of rsTM was measured. After LPS stimulation for 1 h, HUVECs were treated with rsTM for 3 h. Young’s modulus was determined from the force curve (*n* = 5 cells, each group). *p* values were determined by two-way ANOVA with Tukey’s test, * *p* < 0.05; ** *p* < 0.01. The experiments were repeated three independent times with similar results.

Moreover, we found that rsTM suppresses LPS-induced endothelial cellular stiffening in a dose-dependent manner (Figure 2A). Subsequently, we investigated whether post-administration of rsTM reduces the increased EC stiffness induced by LPS stimulation. HUVECs were stimulated with LPS for 1 h and then treated with rsTM for 3 h (Figure S1D). After LPS stimulation for 1 h, HUVECs showed increased cellular stiffness (44.28 ± 6.00 kPa) and maintained their increased stiffness (48.51 ± 8.17 kPa) after 4 h. Interestingly, post-administration of rsTM significantly attenuated the increased endothelial cellular stiffness induced by LPS stimulation (Figure 2B). Thus, these results suggest that rsTM attenuates endothelial cellular stiffness that occurs upon inflammatory stimulation, independently of the inhibition of the LPS-induced activation pathway.

### 3.2. RsTM Improves Cytoskeletal Rearrangement and Focal Adhesion Formation

**Figure 3:**
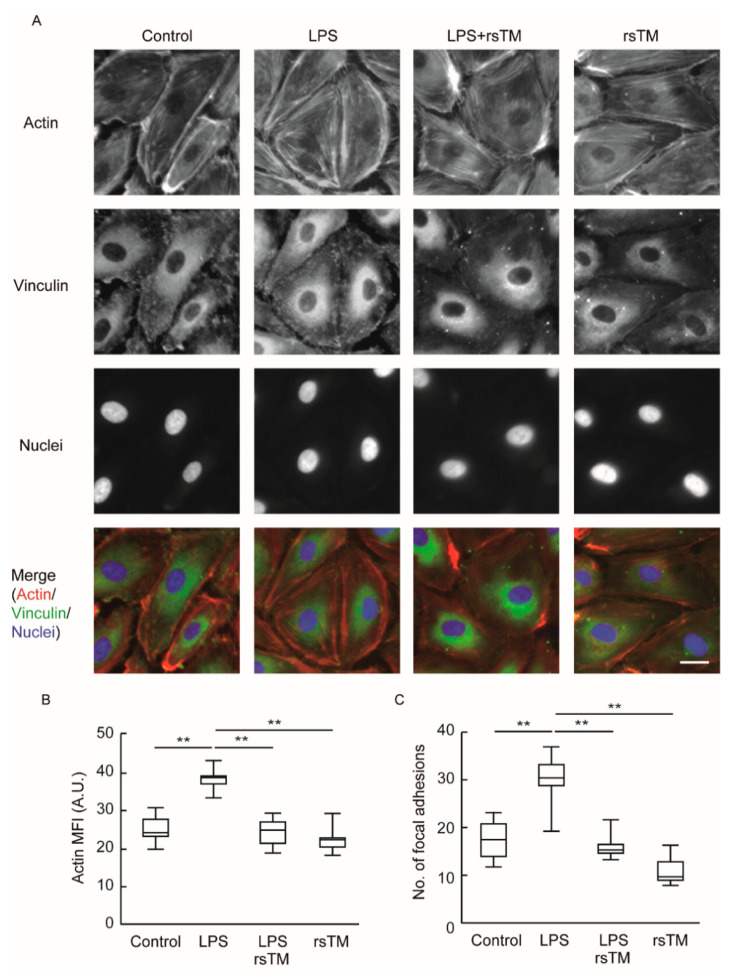
RsTM suppressed LPS-induced stress fiber and focal adhesion formation. (**A**) F-actin and focal adhesion in HUVECs were visualized by fluorescence microscopy. After stimulation with LPS and/or rsTM for 4 h, HUVECs were stained for F-actin using rhodamine-phalloidin, for focal adhesion using anti-vinculin antibody, and for nuclei using DAPI. Merged images of each group are shown. Representative data from three independent experiments are shown. Scar bar showing 20 μm. (**B**) Mean fluorescence intensity (MFI) in a cell was measured using ImageJ software (*n* = 5 cells, each group). (**C**) Number of focal adhesions in a cell were counted using ImageJ software (*n* = 6 cells, each group). *p* values were determined by two-way ANOVA with Tukey’s test, ** *p* < 0.01. The experiments were repeated three independent times with similar results.

Cellular stiffness is predominantly determined by filamentous actin (F-actin) structures and focal adhesion formation [27,28]. As shown in Figure 1D, rsTM suppresses the formation of rigid fiber-like regions in inflamed HUVECs. Thus, we investigated the effects of rsTM on the formation of actin fiber and focal adhesion. The degree of actin fiber and focal adhesion following LPS and/or rsTM treatment was examined by immunofluorescence staining. After LPS stimulation for 4 h, the induction of F-actin stress fiber and increased focal adhesion formation was observed (Figure 3). In contrast, the use of rsTM to treat LPS-stimulated HUVECs suppressed both F-actin structure and focal adhesion formation compared with LPS-stimulated HUVECs. In addition to HUVECs, human aortic endothelial cells also enhance the formation of focal adhesion and stress fibers in response to LPS stimulation, and then reduced them by rsTM treatment (Figure S2).

### 3.3. RsTM Enhances the Gap Junction Functionality of Endothelial Cells

**Figure 4:**
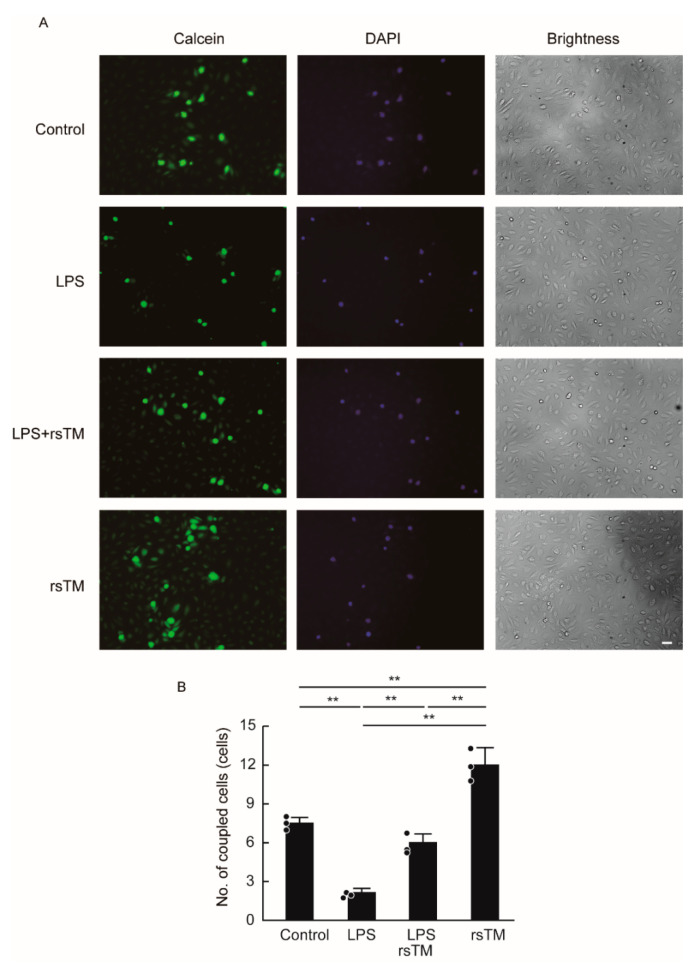
RsTM suppressed the LPS-induced reduction of gap junction functionality in endothelial cells. Non-staining acceptor HUVECs after treatment with LPS and/or rsTM for 4 h were co-cultured with donor HUVECs stained with calcein-AM. (**A**) Evaluation of gap junction functionality in HUVECs. The images of calcein (green), DAPI (nuclei of donor HUVECs, blue), and brightness were observed under fluorescence microscopy. Representative data from three different images are shown. Scar bar showing 40 μm. (**B**) Quantitative evaluation of gap junction functionality in HUVECs. The numbers of dye-migrated acceptor cells per donor cell were calculated in images as shown in Figure 4A (*n* = 3 images, each group). Data are expressed as the means + SD with the circles representing values from individual samples. *p* values were determined by two-way ANOVA with Tukey’s test, ** *p* < 0.01. The experiments were repeated three independent times with similar results.

In addition to increased F-actin structure and focal adhesion formation, the reduction of gap junctions between endothelial cells has also been implicated in the increased endothelial cellular stiffness that occurs during inflammation [15]. We then determined the effect of rsTM on gap junction functionality in LPS-stimulated HUVECs (Figure 4). We stimulated confluent HUVECs with LPS and/or rsTM and then evaluated gap junction functionality by using a dye transfer assay. Calcein-AM generates fluorescence after its digestion by intracellular esterases. Calcein, a small fluorescence dye, transfers to neighboring acceptor cells through an opened gap junction channel, whereas DAPI remains in donor cells in order to bind to DNA. LPS stimulation reduced gap junction functionality in a manner similar to that of TNF-α stimulation [15]. In contrast, rsTM improved the LPS-mediated reduction of gap junction functionality. We confirmed that LPS and/or rsTM treatment did not markedly influence either gap junction protein Cx32 or Cx43 localization (Figure S3). These results suggest that rsTM induces endothelial cellular softening not only by blocking both actin organization and focal adhesion formation but also by enhancing gap junction functionality. Interestingly, rsTM treatment without LPS stimulation enhanced gap junction functionality to a greater degree than in the vehicle-treated control group, suggesting that rsTM improves gap junction functionality independently of the inhibition of the LPS-mediated activation pathway.

### 3.4. Endothelial Cellular Stiffening Promotes Monocyte Adhesion

**Figure 5:**
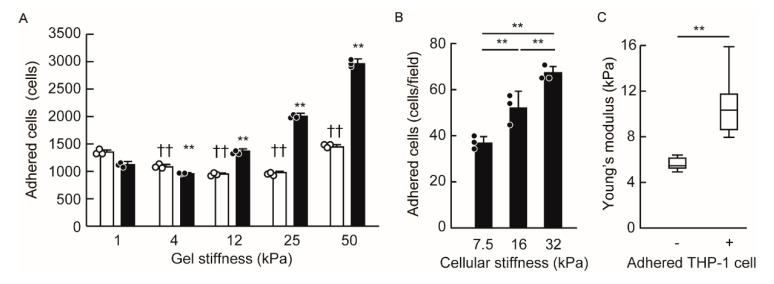
THP-1 cells adhered to the substrate and to the endothelial cells in a stiffness-dependent manner. (**A**) THP-1 cells were stimulated with LPS or vehicle, and were then cultured on ICAM-1-binding gels with the indicated relevant stiffness. After 2 h of incubation, the number of THP-1 cells that stimulated LPS (black column) and vehicle (white column) and adhered to the gels were evaluated by CellTiter-Glo (*n* = 3 biological replicates per group). Data are expressed as the means + SD, with the circles representing values from individual samples. *p* values were determined using a Dunnett’s test, **, ^††^ *p* < 0.01 vs. the number of THP-1 cells on 1 kPa gel with or without LPS stimulation. The experiments were repeated three independent times with similar results. (B) HUVECs were treated with TNF-α and carbenoxolone, blebbistatin, or vehicle. The stiffness measures of HUVECs were 7.5 (TNF-α and blebbistatin), 16 (TNF-α and vehicle), and 32 kPa (TNF-α and carbenoxolone), respectively. After THP-1 cells were co-cultured with HUVECs for 2 h, adhered calcein staining THP-1 cells were counted by fluorescence microscopy (*n* = 3 biological replicates per group). Data are expressed as the means + SD, with the circles representing values from individual samples. *p* values were determined using Tukey’s test, ** *p* < 0.01. The experiments were repeated four independent times with similar results. (C) Measurement of the stiffness of THP-1 cell-adhered HUVECs or non-adhered HUVECs (*n* = 6 cells, each group). *p* values were determined by a Mann–Whitney U test, ** *p* < 0.01. The experiments were repeated two independent times with similar results.

Leukocyte adhesion and activation are promoted by their interaction with a stiff substrate [16,29,30,31]. In order to investigate the impact of substrate stiffness on leukocyte adhesion, we employed ICAM-1-Fc-binding gels (1–50 kPa). We found that these gels, at the same levels, exhibited different degrees of stiffness than normal endothelial cells or inflamed endothelial cells. ICAM-1 is an integrin ligand on endothelial cells and is essential for leukocyte adhesion. The level of ICAM-1-Fc binding on 1 kPa gel was less than that on the other gel; however, within 4–50 kPa gel stiffness, there were no marked differences in terms of the binding of ICAM-1-Fc protein to each gel (Figure S4). Using ICAM-1-Fc-binding gels, we evaluated monocytic THP-1 cell adhesion to each gel. These results showed that monocytic THP-1 cell adhesion increased in a gel stiffness-dependent manner (Figure 5A). Notably, these data indicated that cell adhesion was altered within the range of stiffness that endothelial cells typically exhibit during inflammation.

Next, we investigated whether the stiffness of endothelial cells directly promotes monocyte adhesion. In order to prepare several stiff endothelial cells, we treated HUVECs with the gap junction inhibitor carbeboxolone or the actomyosin II inhibitor blebbistatin in the presence of TNF-α. The stiffness of HUVECs treated with TNF-α alone, TNF-α and carbenoxolone, and TNF-α and blebbistatin presented measurements of 16, 32, and 7.5 kPa, respectively. We then co-cultured THP-1 cells with each endothelial cell in order to examine THP-1 cell adhesion to endothelial cells. The number of adhered THP-1 cells was greater than in stiff endothelial cells, and this occurred in a stiffness-dependent manner (Figure 5B). Moreover, we compared the stiffness of THP-1 cell-adhered and non-adhered HUVECs (Figure S5). Of note, the THP-1 cell-adhered HUVECs showed a greater stiffness than HUVECs without THP-1 cell adhesion (Figure 5C), suggesting that THP-1 cells are more likely to adhere to stiff endothelial cells.

### 3.5. RsTM Suppresses Monocyte Adhesion by Reducing Endothelial Cellular Stiffness

**Figure 6:**
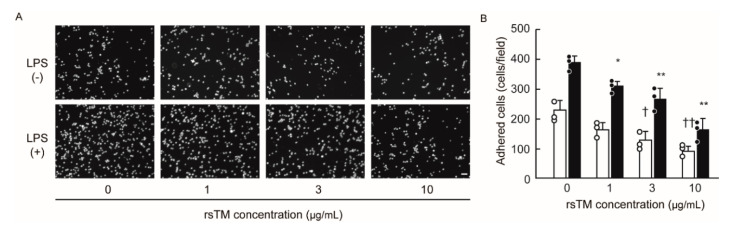
RsTM suppressed THP-1 cell adhesion to LPS-treated HUVECs. HUVECs were treated with (black column) or without (white column) LPS and rsTM (0, 1, 3, 10 μg/mL) for 4 h. After THP-1 cells were co-cultured with HUVECs for 2 h, adhered calcein staining THP-1 cells were observed under fluorescence microscopy. (**A**) Representative data from three independent experiments are shown. Scar bar showing 40μm. (**B**) Adhered THP-1 cells in an image were counted (*n* = 3 images per group). Data are expressed as the means + SD, with the circles representing values from individual samples. The experiments were repeated three independent times with similar results. *p* values were determined using a two-way ANOVA with Tukey’s test, *, ^†^ *p* < 0.05; **, ^††^ *p* < 0.01 vs. the non-rsTM treatment group with or without LPS stimulation.

Next, we determined whether rsTM-induced cellular softening leads to the suppression of monocyte adhesion. In order to exclude the effect of direct interaction between rsTM and leukocyte β2 integrin, we removed rsTM from HUVECs in the supernatant after stimulation, and then co-cultured THP-1 cells with LPS and/or rsTM-treated HUVECs. We evaluated the adhered THP-1 cells by using a cell adhesion assay. The results showed that rsTM-treated endothelial cells diminished the capability of THP-1 cell adhesion, and that this occurred in an rsTM dose-dependent manner (Figure 6). These results suggest that rsTM-induced cellular softening may be involved in the regulation of monocyte adhesion at the inflamed endothelium.

## 4. Discussion

Our previous findings that endothelial cells transiently increased their stiffness upon proinflammatory stimulation suggested that cellular stiffening promotes the progression of vascular inflammation during the acute phase rather than during the chronic phase. In this study, we demonstrated not only that septic LPS-induced sterile inflammation increased endothelial cellular stiffness to the same degree as other proinflammatory stimuli but also that monocytic THP-1 cells are more likely to adhere to stiff HUVECs in a cellular stiffness-dependent manner. The current studies clearly showed that rsTM improves LPS-induced cellular stiffening through the suppression of actin fiber formation and the enhancement of gap junction functionality. Moreover, these studies demonstrate that rsTM suppresses monocyte adhesion by reducing endothelial cellular stiffness. We confirmed that rsTM attenuates the cellular stiffening of HUVECs from different donors and of endothelial cells from arteries upon LPS stimulation, suggesting that the alteration in cellular stiffness might be a common character of endothelial cells. It is well known that LPS stimulation activates endothelial cells and promotes monocyte and leukocyte adhesion to inflamed endothelial cells via the expression of cell adhesion molecules [32], and that rsTM suppresses LPS-mediated endothelial cell activation [10]. In this study, we succeeded in identifying the role of alterations in endothelial cellular stiffness during pathological conditions by using an in vitro LPS-induced inflammation model. These results strongly suggest that rsTM exerts novel anti-inflammatory effects by reducing endothelial cellular stiffness under inflammatory conditions.

Endothelial cells increase their stiffness in response to proinflammatory stimuli, shear stress, oxidized low-lipoprotein, and extracellular environments. Generally, cellular stiffness correlates with actomyosin and myosin light-chain kinase-mediated contraction forces. Thus, their inhibition clearly dampens cellular stiffness [15,33]. Notably, a genetic deficiency or inhibition of myosin light-chain kinase in mice attenuates endothelial permeability, leukocyte transendothelial migration, and atherosclerosis [34]. Cellular stiffness is predominantly determined by cytoskeletal structures, such as the formation of actin bundles, stress fibers, and tensile actomyosin structures, which are promoted by activation of the Rho-actomyosin signaling pathway [27,33,35]. Its activation by proinflammatory stimulation is crucial for endothelial cellular stiffening [36,37]. In addition to proinflammatory stimulation, integrin–extracellular substrate interaction, mechanical stress, and cell–cell junction, all of which contain machineries acting directly in concert with actin cytoskeletal structures, have been implicated in the regulation of cellular stiffness via actin organization [38]. Taken together, these findings suggest that multiple pathways leading to the Rho-actomyosin pathway and actin organization regulate cellular stiffness.

The primary pharmacological action of rsTM stems from its capacity to bind circulating thrombin molecules, thereby serving as an activation complex to convert protein C to activated protein C [1,2]. It has been well-established that rsTM exhibits anti-inflammatory effects by blocking such proinflammatory agonists as LPS, thrombin, HMGB1, and histone. In addition to these mechanisms, several studies have indicated that rsTM directly interacts with G-protein-coupled receptor 15, fibroblast growth factor receptor 1, or unidentified receptors to exert anti-inflammatory cytoprotective effects via inhibition of the mitogen-activated protein kinase signaling cascade and nuclear factor-κ B signaling [10,39]. As these pathways potentially contribute to Rho-actomyosin and actin organization, rsTM might be a potent regulator of cellular stiffness under both inflammatory and normal conditions.

Recent studies strongly suggest that stiff vascular vessels in aging-related arterial stiffening and atherosclerotic plaques promote monocyte adhesion and, subsequently, the progression of atherosclerosis [13,14]. We confirmed that human aortic endothelial cells enhance the formation of focal adhesion and stress fibers (Figure S2), leading to cellular stiffening in response to LPS stimulation, as has similarly been observed with HUVECs. Thus, both artery and vein endothelial cellular stiffening upon exposure to proinflammatory stimuli may trigger leukocyte and monocyte adhesion at inflamed vascular regions. Several studies have shown that leukocyte adhesion and transendothelial migration are facilitated when in contact with stiff vascular vessels or substrates [40,41,42]. This is due to durotaxis, which is recognized as a cell migration property that leads to increased stiffness [43]. Leukocytes sense the stiffness of the extracellular environment via the catch-bond property of integrins and adhere more strongly to stiff substrates [38]. On the other hand, endothelial cells materially work as a substrate that can serve as a critical niche during leukocyte adhesion and migration [38]. Indeed, our data demonstrated that both stiff endothelial cells and gels facilitate THP-1 cell adhesion in an in vitro model. These findings indicate that endothelial cellular stiffness might be a determinant factor of leukocyte adhesion.

The proinflammatory cytokines LPS and HMGB1, as well as extracellular histones, are powerful stimuli that can induce inflammatory responses, including the expression levels of the integrin ligands ICAM-1 and vascular cell adhesion molecule-1 (VCAM-1) on endothelial cells [10]. Indeed, leukocyte adhesion and migration are known hallmarks of vascular inflammation in models of septic mice. It is thought that rsTM suppresses leukocyte adhesion and migration by blocking LPS, HMGB1, and extracellular histones, thereby leading to amelioration of inflammation and organ injury [8,44]. Moreover, we and other groups have shown that rsTM inhibits the adhesion that occurs between leukocytes and endothelial cells by directly blocking integrin–integrin ligand interactions [45,46,47]. Unlike these mechanisms by which rsTM suppresses leukocyte adhesion, the results of the current study provide the first evidence that an rsTM-mediated reduction of endothelial cellular stiffness contributes to the suppression of monocyte adhesion to inflamed endothelial cells.

In addition, proinflammatory stimulation reduces vascular barrier integrity via actomyosin contraction, actin rearrangement, and disruption of intercellular contacts [48]. Additionally, activated protein C, which is generated by the protein C pathway consisting of thrombin, thrombomodulin, and endothelial protein C receptor, improves endothelial vascular barrier function [49,50]. As vascular barrier function is regulated by cytoskeletal rearrangements and cell–cell junctional disorganization, our results suggest the possibility that cellular stiffness is involved in the regulation of vascular barrier function in respond to the state of vascular inflammation and coagulation. Further studies are needed to address how cellular stiffness modulates vascular barrier function and other vascular functions.

Of note, we found that the blockade of gap junctions induces the cellular stiffening associated with focal adhesion formation and cytoskeletal rearrangement [15]. In this study, we demonstrated that rsTM improves gap junction functionality in endothelial cells independently of the inhibition of LPS signaling. Although the mechanisms by which gap junctions mediate the regulation of cellular stiffness remain unclear, the interactions of Cxs with cytoskeletal proteins have been previously described [19]. Chen and colleagues reported that the Cx43 and zona occulden-1 (ZO-1) complex facilitates cell migration via modulation of the F-actin cytoskeleton architecture [51]. In addition, Cx proteins interact with phosphatases and protein kinases, catenins, structural proteins, and microtubules. Cx32 interacts with Src, calmodulin, claudin, occludin, and β catenin [52,53], whereas Cx43 interacts with ZO-1, Src family members, protein kinases, phosphatases, and others [54]. Thus, these studies suggest that gap junctions may regulate cellular stiffness by cytoskeletal rearrangement. In agreement with these finding, our results suggest that the rsTM-induced improvement of gap junction functionality works in conjunction with the remodeling of the actin structure and focal adhesion formation, thereby leading to cellular softening.

Furthermore, the gap junctions and Cx hemichannels present in endothelial cells have been implicated in monocyte adhesion to the endothelium in the context of atherogenesis. Cx40- and Cx37-deleted mice progressively promote development of the atherosclerotic plaques associated with both monocyte and macrophage recruitment [23,24]. Endothelial cells in Cx40-deleted mice increase VCAM-1 expression [24], which is a Mac-1 integrin ligand. Cx37-deficient mice exhibit a number of proinflammatory genes [55], which leads to monocyte accumulation and the development of atherosclerotic plaques. The cellular stiffness-based regulatory mechanism of monocyte adhesion that we describe herein might partly explain the endothelial Cx-related regulation of monocyte adhesion; however, further studies are required to better understand the mechanism by which Cx mediates monocyte adhesion.

Recently, the Sepsis Coagulopathy Asahi Recombinant LE Thrombomodulin (SCARLET) trial, which is the latest multinational multicenter phase III randomized controlled trial, was completed [56]. In this trial study, although rsTM was shown to be biologically active by reducing the plasma levels of the coagulation activation markers D-dimer, prothrombin fragment F1+2, and thrombin-antithrombin complexes, it did not significantly reduce the 28-day mortality rate in patients with sepsis-associated coagulopathy [56]. As the enrolled patient population presented clinical heterogeneity, it has been thought that enrichment of the study population by including patients who may be more likely to benefit from such treatment might be revealing in a future trial. Therefore, elucidation of the anti-inflammatory mechanism underlying rsTM is of increasing importance. Our findings provide insights into the pathogenesis of vascular inflammatory diseases and could be important for the development of antivascular inflammatory therapies targeting cellular stiffness.

## 5. Conclusions

In conclusion, we have clearly shown that rsTM improves LPS-induced cellular stiffening through the suppression of actin fiber formation and the enhancement of gap junction function. In addition, we found that LPS-induced endothelial cellular stiffening facilitates monocyte adhesion. We also revealed that rsTM suppresses monocyte adhesion by reducing endothelial cellular stiffness. Our study proposes another pathway of anti-inflammatory effects: Namely, that rsTM regulates monocyte adhesion through endothelial cellular stiffness, which suggests that rsTM treatment holds some promise for the treatment of vascular inflammatory diseases, such as sepsis.

## Supplementary Materials

The following are available online at https://www.mdpi.com/2073-4409/9/8/1811/s1, Figure S1: Experimental protocols for the measurement of endothelial cellular stiffness., Figure S2: RsTM suppressed LPS-induced stress fiber and focal adhesion formation of aortic endothelial cells., Figure S3: Effect of LPS and rsTM on Cx43 and Cx32 localization in HUVECs., Figure S4: Preparation of ICAM1-Fc coated gels, Figure S5: Measurement of the stiffness of THP-1 cell-adhered and non-adhered HUVECs.
