# Supplementary material for: Recombinant Human Soluble Thrombomodulin Suppresses Monocyte Adhesion by Reducing Lipopolysaccharide-Induced Endothelial Cellular Stiffening
Source: Cells. 2020 Jul 30;9(8):1811. doi: 10.3390/cells9081811 (PMC7463703; doi:10.3390/cells9081811)
Supplement: Supplementary file 1 [file cells-09-01811-s001.pdf]

Figure S1

Okamoto, T. et al.

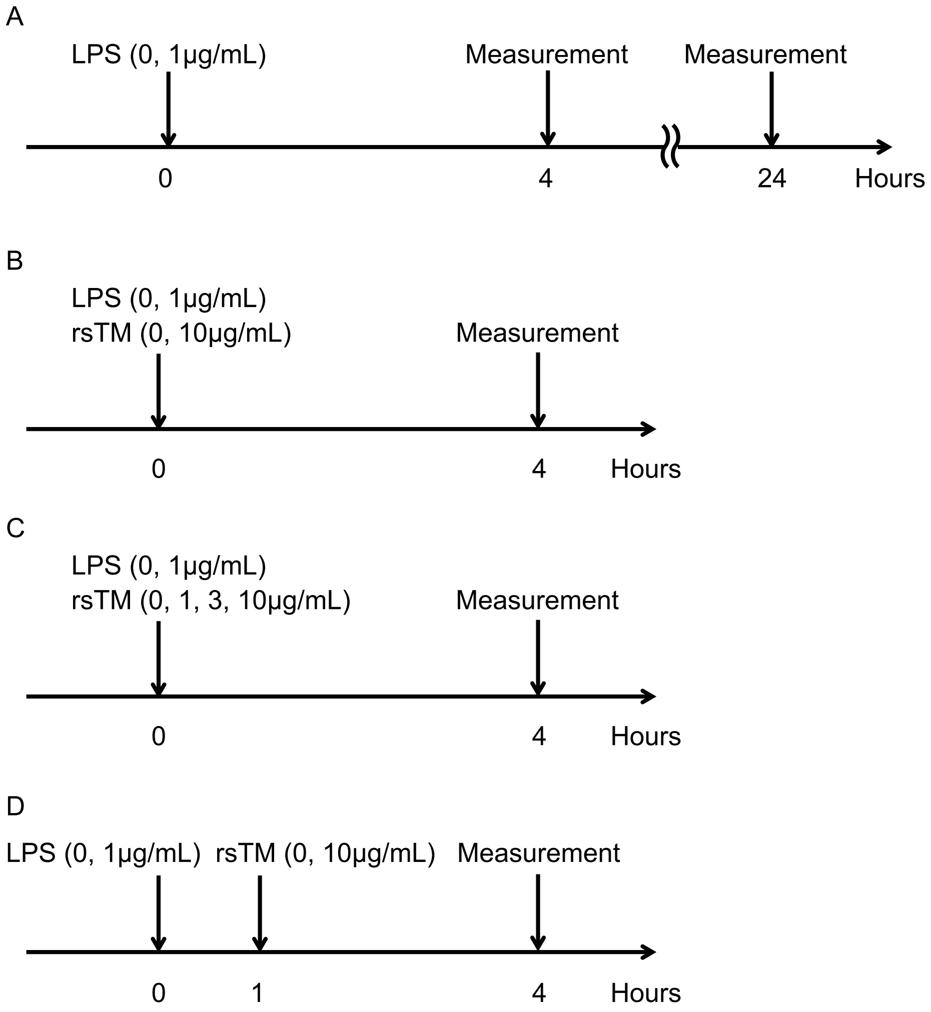

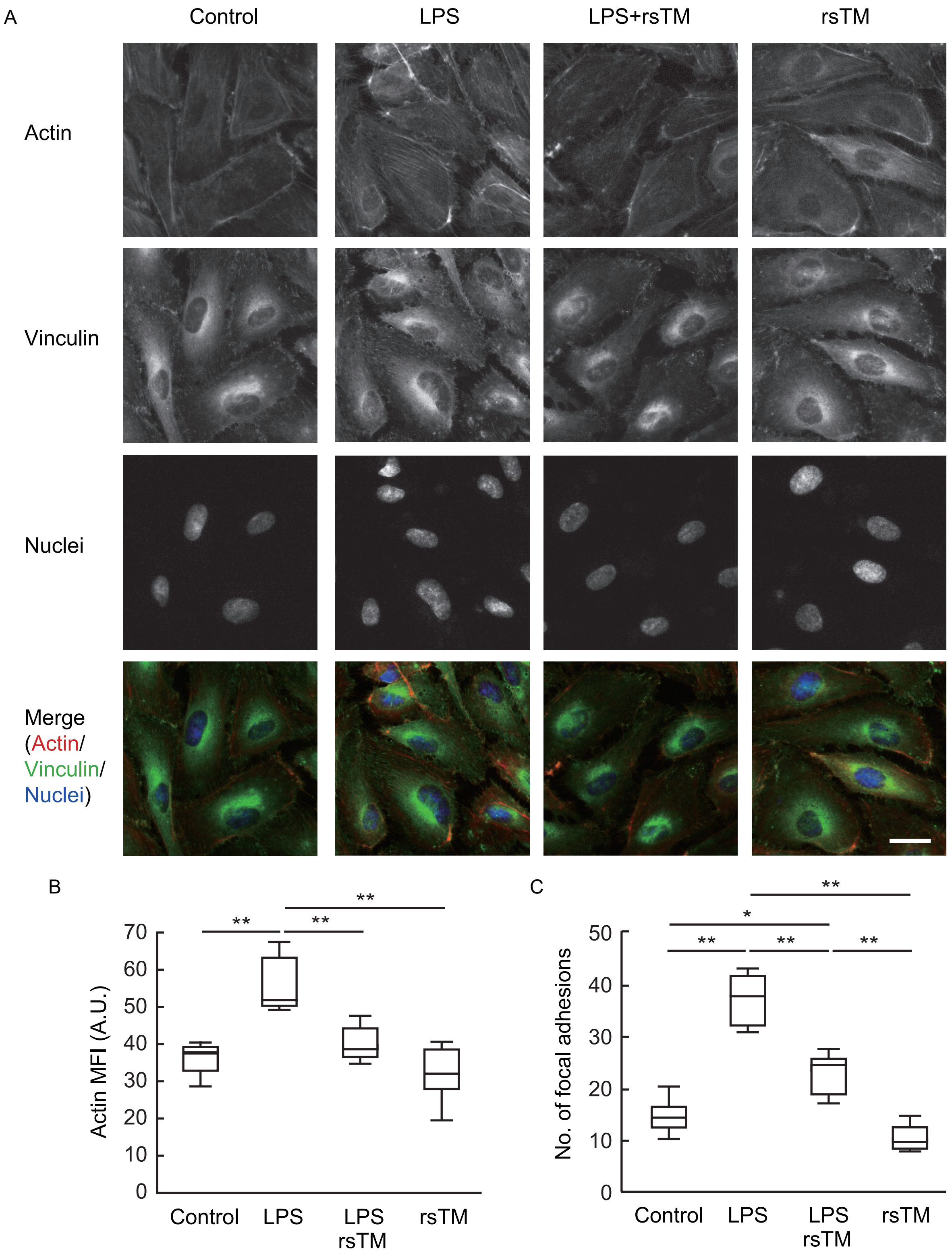
A
Control
LPS
LPS+rsTM
rsTM
Actin
Vinculin
Nuclei
Merge
(Actin/
Vinculin/
Nuclei)
B
Actin MFI (A.U.)
70
60
50
40
30
20
10
0
**
Control
LPS
LPS
rsTM
rsTM
C
No. of focal adhesions
50
40
30
20
10
0
*
**
Control
LPS
LPS
rsTM
rsTM

Figure S3
Okamoto, T. et al.

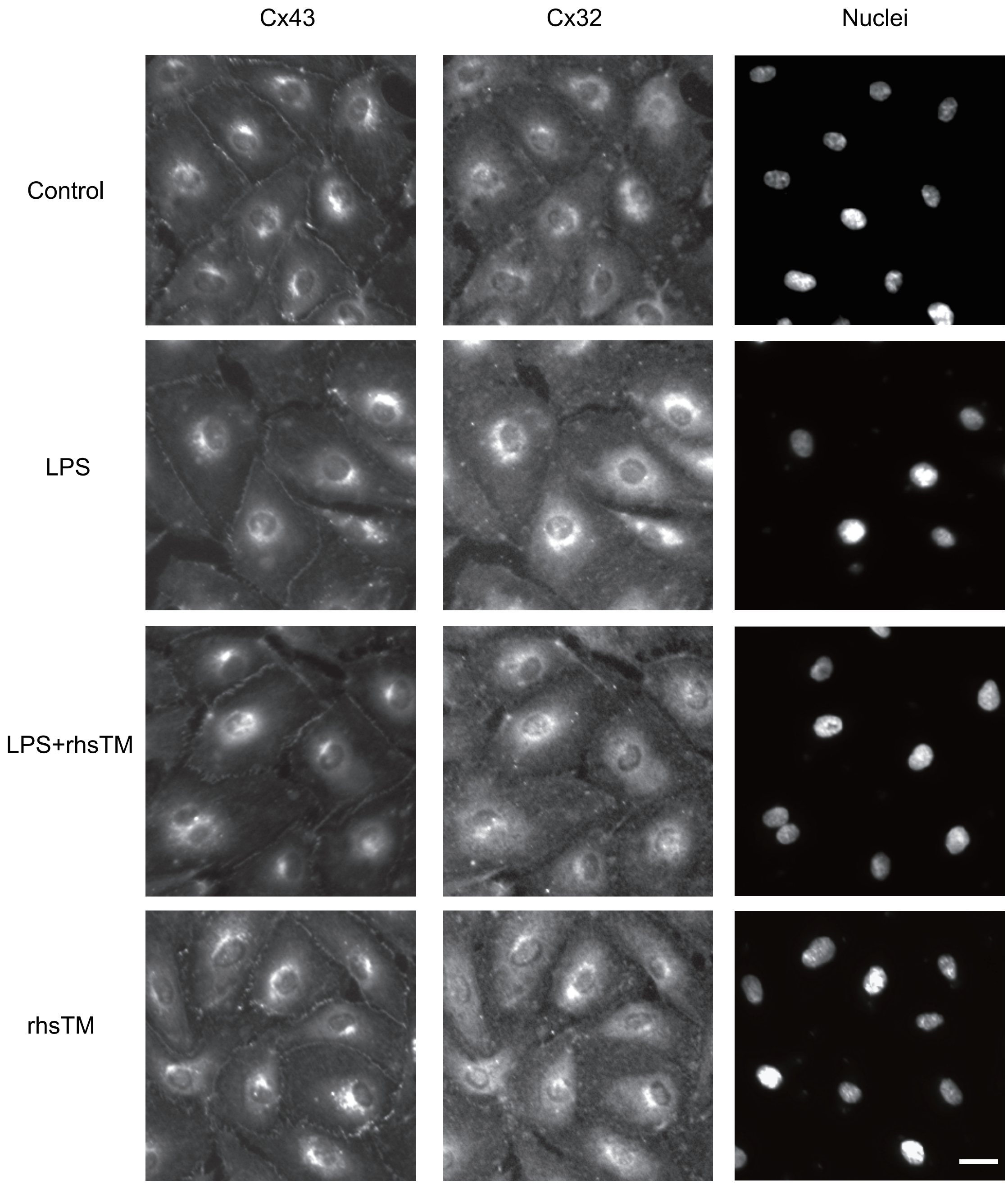

Figure S4
Okamoto, T. et al.

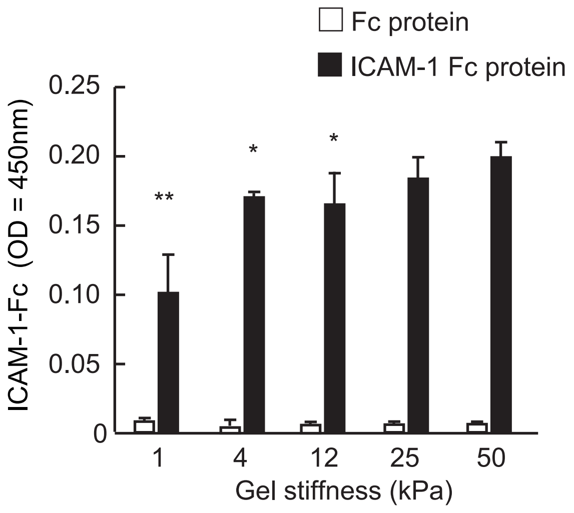

Figure S5

Okamoto, T. et al.

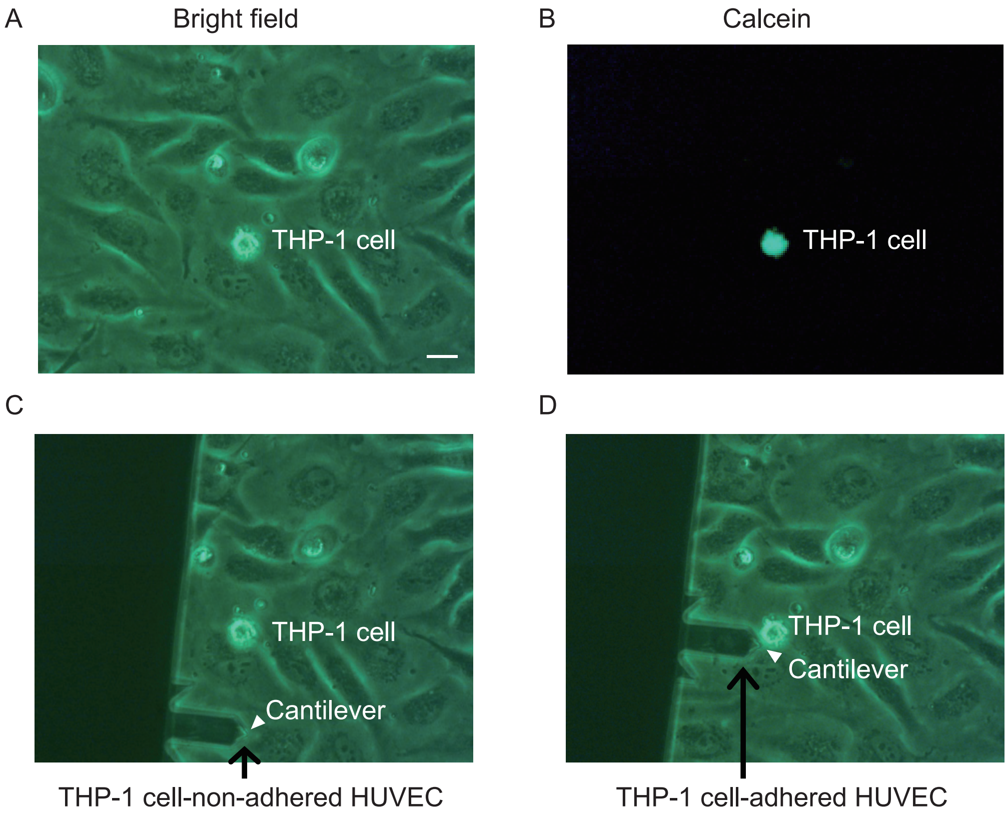

Supplementary files

Figure S1. Experimental protocols for the measurement of endothelial cellular stiffness. (A) Measurement of cultured endothelial cellular stiffness after 4 hours and 24 hours of LPS stimulation. (B) Measurement of cultured endothelial cellular stiffness after 4 hours of LPS stimulation and/or rsTM administration. (C) Dose-dependent effect of rsTM on LPS-induced endothelial cellular stiffening. (D) The effect of post-administration of rsTM on LPS-induced endothelial cellular stiffening.

Figure S2. RsTM suppressed LPS-induced stress fiber and focal adhesion formation of aortic endothelial cells.

(A) F-actin and focal adhesion in HAECs were visualized by fluorescence microscopy. After stimulation with LPS and/or rsTM for 4 hours, HAECs were stained for F-actin using rhodamine-phalloidin, for focal adhesion using anti-vinculin antibody, and for nuclei using DAPI. Merged images of each group are shown. Representative data from 5 pictures are shown. Scar bar showing 23 μm. (B) Mean fluorescence intensity (MFI) in a cell was measured using ImageJ software (n = 5 cells, each group). (C) The number of focal adhesions in a cell

were counted using ImageJ software (n = 5 cells per group). P values were determined by two-way ANOVA with Tukey's test, $*P < 0.05$; $**P < 0.01$. The experiments were repeated two independent times with similar results.

Figure S3. Effect of LPS and rsTM on Cx43 and Cx32 localization in HUVECs. The functionality of the gap junctions formed by endothelial Cx43 and Cx32 is implicated in the regulation of endothelial cellular stiffness. To investigate Cx43 and Cx32 protein localization, HUVECs were treated with LPS and/or rsTM and then stained with anti-Cx43 antibody, anti-Cx32 antibody, and DAPI. Cx43 and Cx32 protein localization did not become altered after LPS stimulation and rsTM treatment. These data indicated that the typical expression patterns of both Cx43 and Cx32 proteins in healthy endothelial cells were apparent in all groups. Representative data from three independent experiments are shown. Scar bar showing 20μm.

Figure S4. Preparation of ICAM1-Fc coated gels. Chemically activated poly-acrylamide hydrogels (1, 4, 12, 25, or 50 kPa) in 24-well plates were incubated with recombinant human ICAM-1-Fc (5 μg/mL: black column) or recombinant Fc

(5 μg/mL: white column) for 1 hour and then blocked with BSA. The amounts of binding ICAM-1-Fc to the gel were detected via an enzyme immunoassay assay using primary anti-ICAM-1 monoclonal antibody and secondary anti-mouse IgG-horseradish peroxidase conjugate (n = 3 biological replicates per group). Color development was obtained for horseradish peroxidase by adding 100 μL of tetramethylbenzidine substrate solution. The reaction was then stopped with 100 μL of 2 N H_2SO_4. The absorbances were measured at 450 nm. *P* values were determined with a Dunnett's test, $^{*,\dagger}P< 0.05$; $^{**,\dagger\dagger}P< 0.01$ vs 50 kPa gel group incubated with ICAM-1-Fc or recombinant Fc.

Figure S5. Measurement of the stiffness of THP-1 cell-adhered and non-adhered HUVECs. HUVECs were co-cultured with calcein-labeled THP-1 cells for 1 hour. After washing, cells were observed under fluorescence microscopy. Panel A-D showing the same area. (A) A bright field showed the HUVEC monolayer and the adherent THP-1 cell. (B) THP-1 cells were identified by detection of calcein dye. The stiffness of THP-1 cell non-adhered (C) and adhered (D) HUVECs (arrow) was determined by a cantilever as indicated (white arrow head).
